# Effects of Red Clover Isoflavones on Growth Performance, Immune Function, and Cecal Microflora of Mice

**DOI:** 10.3390/ani15020150

**Published:** 2025-01-09

**Authors:** Rongrong Guo, Xuqin Song, Xiaodie Li, Cheng Zeng, Ying Chen, Chunjie Li, Jian Yang, Deyuan Ou

**Affiliations:** 1 Department of Veterinary Medicine, College of Animal Science, Guizhou University, Guiyang 550025, China; gs.rrguo22@gzu.edu.cn (R.G.); xqsong@gzu.edu.cn (X.S.); gs.xdli22@gzu.edu.cn (X.L.); ce.czeng20@gzu.edu.cn (C.Z.); yingchen1114@163.com (Y.C.); 2 Laboratory of Pulmonary and Inflammation, Frontiers Science Center for Disease-Related Molecular Network, Sichuan University, Chengdu 610000, China; chunjieli@wchscu.cn

**Keywords:** red clover isoflavones, growth promotion, blood biochemistry, immune function, cecal microflora

## Abstract

Isoflavone components extracted from red clover have anti-inflammatory, antioxidant and immune boosting effects. We hypothesize that red clover isoflavones (RCIs) achieve health-promoting effects via altering the gut microbiota. A total of 48 mice (20 ± 2 g) were randomly divided into a control group, low-dose group (0.05% RCIs in feed), middle-dose group (0.1% RCIs in feed), and high-dose group (0.2% RCIs in feed) with 12 mice per group. The feeding period was 20 d. The results showed that RCIs can increase the daily gain and decrease the ratio of feed to gain in mice. The organ indexes and blood biochemical indexes of the mice in each RCI group were in the normal range, indicating that RCIs do not damage liver or kidney function. RCI supplementation increased serum immunity and altered the microbial community structure in the cecum of the mice. RCIs can increase the diversity of beneficial bacteria such as *Bacteroidaceae*, *Muribaculaceae*, and *Akkermansiaceae*, and reduced the pathogenic *Staphylococcaceae*. Therefore, supplementing the diet with RCIs results in improved growth performance and notable alterations in the cecal microbiota in mice, and has potential applications as a feed additive to improve livestock production.

## Simple Summary

Developing new alternatives to antimicrobials to promote growth, health, and feed efficiency can provide new ideas for the sustainable development of the livestock and poultry industry. In this study, we have documented that adding 0.1% Chinese herbal red clover isoflavones exhibited the advantages of dramatically accelerating animal growth, boosting immunity, and changing the cecal microbiota structure. In particular, potential pathogenic bacteria were successfully decreased and beneficial bacterium in cecal microflora were boosted by adding 0.1% red clover isoflavones to the feed. This study provides a theoretical basis for the practical application of red clover isoflavones as “green” feed additives in animal production.

## 1. Introduction

As the breeding sector evolves, pig farming has emerged as a significant component of the agricultural economy. Nonetheless, piglets encounter pathological and environmental obstacles throughout their development, rendering them susceptible to diarrhea, stunted growth and elevated mortality rates, which negatively impact the development of the swine industry [1]. The breeding industry in our country has entered an era of reducing resistance and preventing resistance. Since the implementation of the antibiotic feed additive withdrawal plan in 2020, the development of novel feed additives to enhance growth and improve the immunity of swine has emerged as a significant focus within the breeding industry. The feed additive that is derived from Chinese herbal medicine has the potential to have a beneficial impact on the growth, immunity, resistance to illness, breeding, and production performance of livestock and poultry. At the same time, it has a low toxicity, with the absence of drug residues, and it has a significant potential to replace antibiotics.

Isoflavones are a group of polyphenolic compounds that are abundant in plants and have attracted significant attention in recent years. Chen et al. [2] reported that soy isoflavone has the potential to regulate the intestinal microbiota, inhibit the development of pathogenic bacteria, and promote the growth of *Bifidobacterium* and *Lactobacillus* probiotics. Genistein can improve gastrointestinal dysfunctions, including intestinal inflammation, intestinal permeability, and epithelial injury [3]. After treating with genistein, the structure of the gut microbiota changed dramatically through increases in the abundance of *Lachnospira* and the production of more short chain fatty acids. Liu et al. [4] reported that puerarin increased the abundance of *Lactobacillaceae*, *Lachospiraceae*, and *Rikenellaceae*, which is beneficial to alleviate oxidative stress and inflammation, thus further postponing aging.

*Trifolium pratense* L., as leguminous plants of the genus clover, are widely distributed in Xinjiang, Guizhou, Yunnan, and many regions in China. Red clover isoflavones (RCIs), the primary active component of red clover, have many pharmacological effects, such as anti-inflammatory, antioxidant, and estrogen-like effects [5]. Research indicates that RCIs can reduce the expression of inflammation-related genes and the secretion of inflammatory cytokines in an LPS-induced 3D4/2 cell inflammation model through modulating the NF-κB and MAPK inflammatory pathways [6]. Another research article exhibited that biochanin A in RCIs may markedly relieve chronic injury in rats caused by carbon tetrachloride and oxidative stress [7]. Feeding RCIs to cows can enhance the activity of antioxidant enzymes including catalase (CAT), glutathione peroxidase (GSH-Px), and superoxide dismutase (SOD), and can enhance the total antioxidant capacity (T-AOC) in serum. Moreover, antioxidant milk from RCIs could prevent oxidative stress and intestinal injury in mice [8]. When RCIs served as a vaccination adjuvant, they significantly enhanced the viability and cytokine expression of spleen cells. Interleukin (IL)-2, interferon-γ (IFN-γ), and IL-10 levels were elevated, and the mRNA expression levels of IL-2, IFN-γ, IL-4, and IL-10 also showed a similar trend when compared with the blank group [9]. Biochanin A in RCIs has an inhibiting impact on *Methicillin-resistant Staphylococcus aureus* (MRSA) and can protect the host through reducing the release of hemolysin from MRSA [10]. In addition, adding RCIs to the daily diet can delay neurodegeneration in the brain and offer a protective impact on neurons, thereby reducing the incidence of Parkinson’s disease in high-risk individuals [11]. The majority of studies emphasize the anti-inflammatory, antioxidant, antibacterial, and neuroprotective properties of RCIs, while there are few studies about their regulation of enteric microorganism.

Today, more and more studies have shown that that microbial flora can improve health by enhancing digestion and immunity [12]. Supplementation with Xiasangju residues can enhance the relative abundance of two prominent probiotics, *Lactobacillus johnsonii* and *Weissella jogaeotgali*, while diminishing the relative abundance of *Escherichia coli*. This may be a principal factor by which Xiasangju residues ameliorate diarrhea and fortify the intestinal barrier in piglets [13]. Another study has proven that the growth performance of broilers was related to the cecal microbiota composition [14]. Some bacteria such as *Ochrobactrum*, *Odoribater*, *Klebsiella*, *Enterobacter*, *Georgenia*, and *Bifidobacterium* can enhance immunity through increasing immunoglobulin G (IgG), immunoglobulin M (IgM), and immunoglobulin A (IgA) levels [15].

Although several studies have proven that RCIs promote animal immunity, there is a lack of literature addressing their potential to promote animal growth and improve the intestinal microbiota. Therefore, this study examined the growth-promoting effects of RCIs in mice, along with blood biochemical indicators, immunological function, and the impact on cecal microflora, which will provide a theoretical foundation for the use of RCIs as a feed supplement in the application of animal raising.

## 2. Materials and Methods

### 2.1. Animals and Experimental Design

The Guizhou University Subcommittee of Experimental Animal Ethics gave approval to all procedures. A total of 48 specific-pathogen-free (SPF) Kunming mice (4 weeks, 18–22 g) were purchased from Henan Skobase Biotechnology Co., Ltd., Anyang, China (SCXK (Yu)2020-0005). For a 30-day feeding period, mice were separated into 4 groups of 12 mice each (equally divided by sex), with the first 10 days being employed for acclimatization. Mice were weighed on the first day and again on the twentieth day. The control group of mice received a baseline diet and Table 1 lists the components and nutritional values. In the RCI additive groups, 0.05%, 0.1%, and 0.2% RCIs were supplemented to basic diet, accordingly.

### 2.2. Growth Performance Analysis

For each group, the average daily feed intake (ADFI), average daily gain (ADG), and feed to gain ratio (F/G) were computed using the following formula:

ADFI = total feed consumption per column/(days of animal testing × number of mice per column);(1)

ADG = (final mean weight − initial mean weight)/trial days;(2)

F/G = ADFI/ADG(3)

### 2.3. Determination of Blood Physiological and Serum Biochemical Parameters

At 24 h after the last feeding RCI, mice were measured for weight and anesthetized with isoflurane. Blood samples were collected and routine hematological indexes were assessed using an automated blood cell analyzer for animals (Shenzhen MINDRAY biological medical electronic Limited by Share Ltd., Shenzhen, China). Blood samples were kept at room temperature before centrifugation at 3000 rpm for 15 min at 4 °C. Then, the upper layer (serum) was collected and kept at −20 °C until it needed to be used. Total protein (TP), alkaline phosphatase (ALP), and aspartate aminotransferase (AST) were detected by automatic biochemical analyzer (Shenzhen MINDRAY biological medical electronic Limited by Share Ltd., Shenzhen, China). Lactate dehydrogenase (LDH), creatine kinase (CK), albumin (ALB), blood urea nitrogen (BUN), and alanine aminotransferase (ALT) were analyzed with corresponding kits (Nanjing Jiancheng Bioengineering Institute, Nanjing, China).

### 2.4. Measurement of Organ Indexes and Immune Indicators

The heart, liver, spleen, lung, and kidney of mice were collected and weighed. The organ indexes were calculated as the organ weight (mg)/body weight (g). The levels of IgG, IgM, IgA, interferon-γ (IFN-γ), complement C3 (C3), complement C4 (C4), and lysozyme (LZM) were assayed with corresponding kits (Nanjing Jiancheng Bioengineering Institute, Nanjing, China).

### 2.5. Microbiological Analysis

A dry, sterile tube was applied to collect the cecal contents of mice, which were then frozen in liquid nitrogen for 10 min and stored in a refrigerator set at −80 °C. After being collected and fixed, cecal tissues were embedded by paraffin and cut into 3 μm thick slices. Finally, slices were stained with hematoxylin and eosin (H&E) for light microscopy analysis. The mouse cecal contents were analyzed by high-throughput sequencing analysis of the V4 region of 16S rDNA. The 16S ribosomal DNA (rDNA) genes of distinct regions (16S V3–V4) were amplified using the following specific primers (341F [5′-CCTAYGGGRBGCASCAG-3′] and 806R [5′-GGACTACNNGGGTATCTAAT-3′] with the barcode. The PCR amplification system consisted of 15 µL of Phusion^®^ High-Fidelity PCR Master Mix (New England Biolabs, Beverly, MA, USA), 0.2 µM forward and reverse primers, and 10 ng of template DNA. After starting the PCR at 95 °C for 2 min, there were 27 cycles of 98 °C for 10 s, 62 °C for 30 s, 68 °C for 30 s, and a final extension at 68 °C for 10 min. PCR amplicons were identified using electrophoresis with 2% agarose gel and purified using the Universal DNA Kit (TianGen Biotech, Beijing, China). The TruSeq^®^ DNA PCR-Free Sample Preparation Kit (Illumina, San Diego, CA, USA) was used for building the sequencing libraries. The Agilent Bioanalyzer 2100 system and Qubit @2.0 Fluorometer (Thermo Scientific, Waltham, MA, USA) were used to evaluate the accuracy of the libraries. Ultimately, a 250 bp paired-end sequence was obtained by sequencing the library using an Illumina NovaSeq instrument.

Original sequences were imported and processed according to the barcode sequence and PCR primer sequence. To obtain high-quality original readings, fastp software (v0.22.0, https://github.com/OpenGene/fastp (accessed on 8 April 2024)) was used. To acquire high-quality tag data, FLASH software (v1.2.11, http://ccb.jhu.edu/software/FLASH/ (accessed on 12 April 2024)) was used. Finally, chimera sequence was removed to get effective data by using vsearch (v2.22.1, https://github.com/torognes/vsearch/ (accessed on 15 April 2024))) and species database. R software (v4.2.0) was used to analyze the sample rationality and species diversity of species dilution curve and rank abundance curve. Chao1, ACE, Simpson, and Shannon indexes were used for α diversity analysis. Primarily, principal coordinate analysis (PCoA) served to examine the β variety. Analysis of species diversity and composition was carried out at the family and phylum levels. To statistically assess group differences, we used linear discriminant analysis (LDA) effect size (LEfSe) techniques.

### 2.6. Statistical Analysis

The statistical analysis was carried out using SPSS version 26.0. All data were presented as mean ± standard deviation (SD). Variations in measurements within groups were determined using one-way analysis of variance. LSD, Duncan and Waller–Duncan post-hoc tests were used for multiple comparisons analysis. A *p*-value of less than 0.05 was considered a significant difference, while a *p*-value of less than 0.01 was considered a highly significant difference.

## 3. Results

### 3.1. Impact of RCIs on Mice’s Growth Performance, Serum Physiological Responses, and Biochemical Indicators

**Figure 1:**
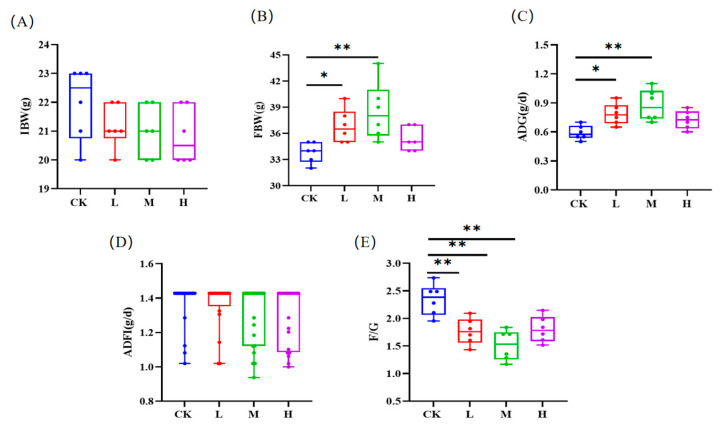
The effects of red clover isoflavones (RCIs) on the growth performance of mice: (**A**) initial body weight (IBW); (**B**) final body weight (FBW); (**C**) average daily gain (ADG); (**D**) average daily feed intake (ADFI); and (**E**) feed to gain ratio (F/G). A basic diet was given to the mice in the control group and L, M, H group were fed with 0.05%, 0.1%, and 0.2% RCI in a basic diet, respectively. * *p* < 0.05; ** *p* < 0.01.

Figure 1A,D shows that the initial body weight (IBW) and ADFI of the mice had no significant differences. The ADG of the mice in the L, M, and H groups were significantly increased by 32.20%, 47.46%, and 23.73%. The mice in each RCI group exhibited greater final body weights (FBWs) and ADGs than the control group, with the middle RCI group reaching the greatest value, indicating a significant difference (*p* < 0.01; Figure 1B,C). Moreover, the F/G ratios in the RCI groups decreased by 24.79%, 35.47%, and 23.08%, respectively. Compared with the control group, the F/G ratios in the RCI groups were significantly lower (*p* < 0.01; Figure 1E).

**Figure 2:**
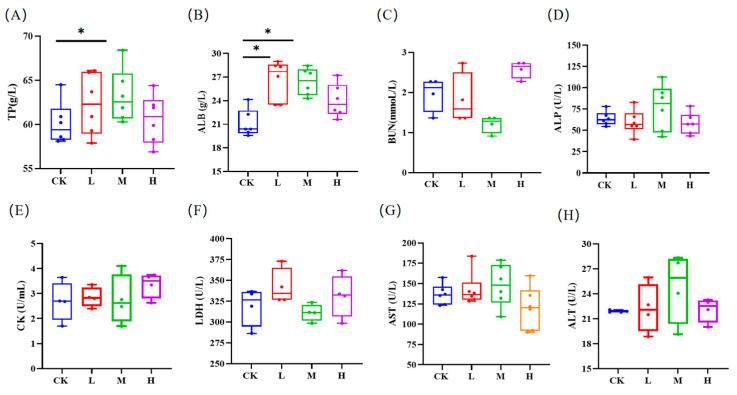
The impact of red clover isoflavones (RCIs) on the blood biochemical and physiological parameters of mice: (**A**) total protein (TP); (**B**) albumin (ALB); (**C**) blood urea nitrogen (BUN); (**D**) alkaline phosphatase (ALP); (**E**) creatine kinase (CK); (**F**) lactate dehydrogenase (LDH); (**G**) aspartate aminotransferase (AST); and (**H**) alanine aminotransferase (ALT). The meanings of CK, L, M, and H are defined as shown in Figure 2. * *p* < 0.05.

Table 2 lists the routine blood indexes of the mice. The white blood cell (WBC) level of the mice in the low and middle RCI groups exhibited drastically higher values than that in the control group (*p* < 0.05). The low RCI group exhibited significantly more neutrophils (Neu#) than the control group (*p* < 0.05). In comparison to the control group, lymphocyte (Lym#) and monocyte (Mom#) numbers in middle RCI group were statistically higher (*p* < 0.05). Figure 2A shows that the TP level in the middle RCI group increased significantly (*p*< 0.05). Significant rises in ALB were observed in the low and middle groups in comparison to the control group (*p* < 0.01; Figure 2B). Nevertheless, no significant differences were observed in the levels of BUN, ALP, CK, LDH, AST, and ALT (*p* > 0.05; Figure 2C–H).

### 3.2. Determination of Organ Indexes and Immune Indicators

**Figure 3:**
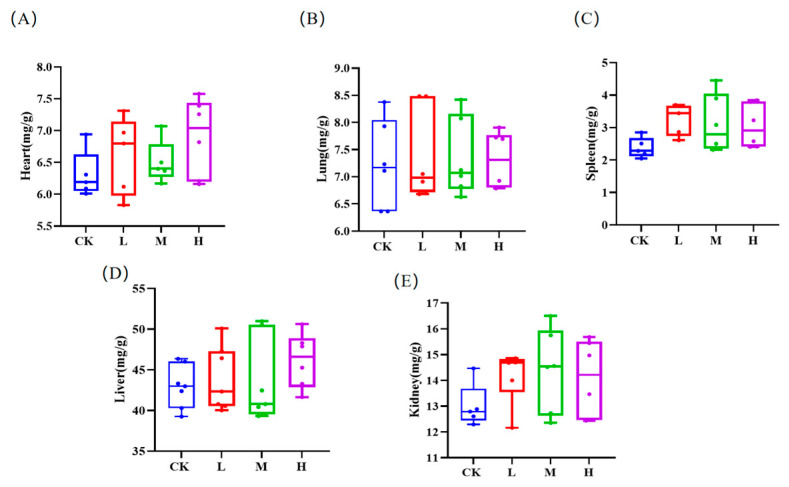
The effects of red clover isoflavones (RCIs) on the organ indicators of mice: (**A**) Heart; (**B**) Lung; (**C**) Spleen; (**D**) Liver; and (**E**) Kidney. The meanings of CK, L, M, and H are defined as shown in Figure 3.

**Figure 4:**
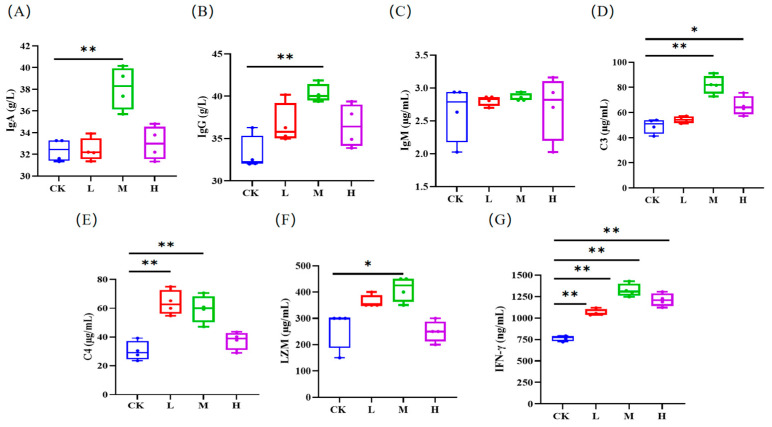
The influence of red clover isoflavones (RCIs) on the serum immune indicators of mice: (**A**) immunoglobulin A (IgA); (**B**) immunoglobulin G (IgG); (**C**) immunoglobulin M (IgM); (**D**) complement C3 (C3); (**E**) complement C4 (C4); (**F**) lysozyme (LZM); and (**G**) interferon-γ (IFN-γ). The meanings of CK, L, M, and H are defined as shown in Figure 4. * *p* < 0.05; ** *p* < 0.01.

Although the organ indexes of the mice in the RCI groups were typically higher than those in the control group, there was no significant difference (*p* > 0.05, Figure 3). As shown in Figure 4A,B,F, IgA, IFN-γ, and LZM levels in the middle RCI group were increased remarkably (*p* < 0.05). The C3 levels in the middle and high RCI groups was dramatically higher than in the control group (*p* < 0.05, Figure 4D). There was a substantial increase in both C4 and IFN-γ levels in each RCI group (*p* < 0.01; Figure 4E,G).

### 3.3. Influence of RCIs on the Cecal Structure and Microbiota of Mice

#### 3.3.1. Cecal Tissue Observation

**Figure 5:**
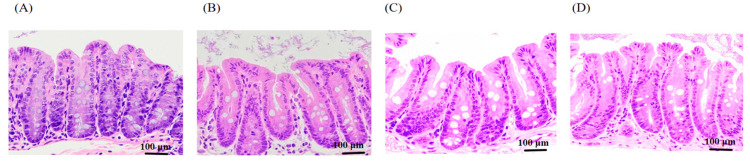
The effects of red clover isoflavones (RCIs) on the pathological changes in the cecal tissue of mice: (**A**) control group (CK); (**B**) 0.05% RCIs (L); (**C**) 0.1% RCIs (M); (**D**) 0.2% RCIs (H). The meanings of CK, L, M, and H are defined as shown in Figure 5.

Compared with the control group, the cecal villi of the mice in the RCI groups exhibited an orderly arrangement, with no interstitial edema, no inflammatory cell infiltration, a normal tissue structure, and no significant histological lesions (Figure 5).

#### 3.3.2. Effects of RCIs on the Cecal Microbiota of Mice

**Figure 6:**
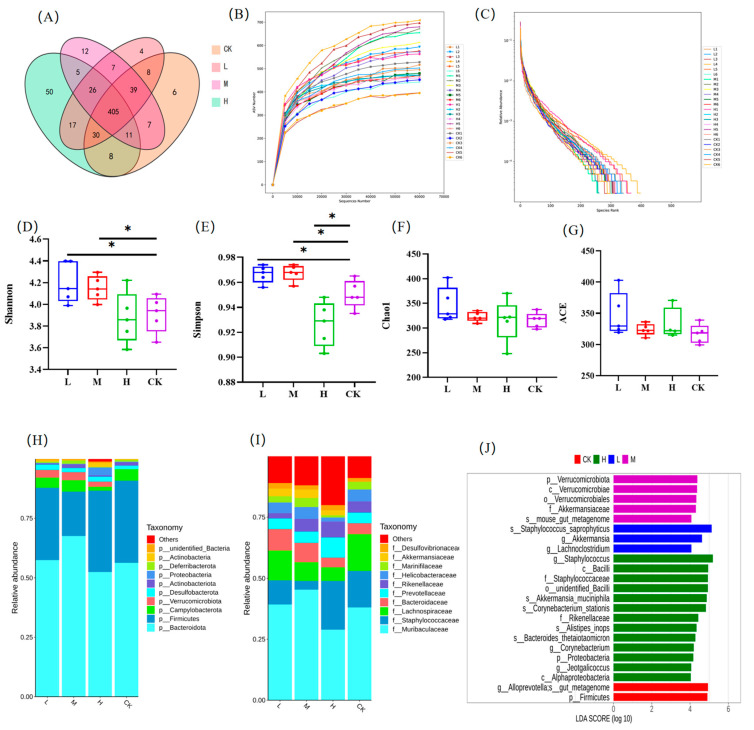
The effect of red clover isoflavones (RCIs) on the cecal microbiota of mice: (**A**) Venn diagram based on ASV; (**B**) Rarefaction Curve for each sample based on ASV; (**C**) Rank Abundance for each sample based on ASV; (**D**) Shannon index; (**E**) Simpson index; (**F**) Chao1 index; (**G**) ACE index; (**H**) relative abundances of phylum; (**I**) relative abundances of family; and (**J**) cladogram graphic that displays the findings for the LDA effect size (LEfSe). Significant differences were defined as taxa with an LDA > 4.0 and *p* < 0.05; the prefixes “p” and “f” indicate the annotated phylum and family levels, accordingly. The meanings of CK, L, M, and H are defined as shown in Figure 6. * *p* < 0.05.

There were 585 genuine ASVs, with the counts for all groups being 514, 536, 512, and 552, respectively (Figure 6A). An analysis and comparison across groups demonstrated that the Rarefaction Curve directly indicates the veracity of the sequencing data and indirectly represents the species richness of the sample. As the curve approaches a plateau, it verifies a growing volume of sequencing data. The rank abundance intuitively signifies the diversity and uniformity of the species within a sample. In the horizontal dimension, species richness is shown by the curve’s breadth; more species richness corresponds to a wider curve. In this study, the sequencing depth is sufficient and contains most species, providing an adequate amount of sequencing data and species richness in all analyzed samples to facilitate additional research (Figure 6B,C). The Simpson indexes in the low and middle RCI groups were significantly greater than in the control group (*p* < 0.05, Figure 6D,E), while the Chao1 and ACE levels exhibited no significant changes (*p* > 0.05, Figure 6F,G).

At the phylum level, *Bacteroidota*, *Firmicutes*, *Campylobacterota*, *Verrucomicrobia*, *Desulfobacterota*, *Actinobacteriota*, *Deferribacterota*, *Actinobacteria*, *Unidentified Bacteria*, and *Proteobacteria* were the 10 most abundant phyla in the cecum of the mice (Figure 6H). *Bacteroidota* and *Firmicutes* were the predominant phyla and the most prevalent bacteria at the phylum level. The species richness of *Bacteroidota* had risen (*p* > 0.05), whereas *Firmicutes* drastically diminished in the low and middle RCI groups (*p* < 0.01). Accordingly, the *Firmicutes* to *Bacteroidota* (F/B) ratio was low (*p* < 0.05). Notably, *Verrucomicrobia* were enhanced in each RCI group compared to the control group.

At the family level, *Muribaculaceae*, *Staphylococcaceae*, *Lachnospiraceae*, *Bacteroidaceae*, *Rikenellaceae*, *Helicobacteraceae*, *Prevotellaceae*, *Marinifilaceae*, *Ruminococcaceae*, and *Akkermansiaceae* were the 10 families with the most richness (Figure 6I). The relative abundances of *Muribaculaceae* and *Bacteroidaceae* were significantly higher (*p* < 0.05), while *Staphylococcaceae* revealed the opposite trend in middle group. *Akkermansiaceae* were enhanced in each RCI group compared to the control group.

The LDA histogram reveals that 23 biomarkers had LDA scores > 4, with 5, 3, 13, and 2 dominant taxa in the cecal samples from the middle, low, high, and control groups, respectively (Figure 6J). At the phylum and family levels, in the medium group, *Verrucomicrobia* and *Akkermansiaceae* were abundant. In the high RCI group, *Proteobacteria*, *Staphylococcaceae*, and *Rikenellaceae* were more abundant, whereas *Firmicutes* was the dominant bacteria.

#### 3.3.3. Spearman Correlation

**Figure 7:**
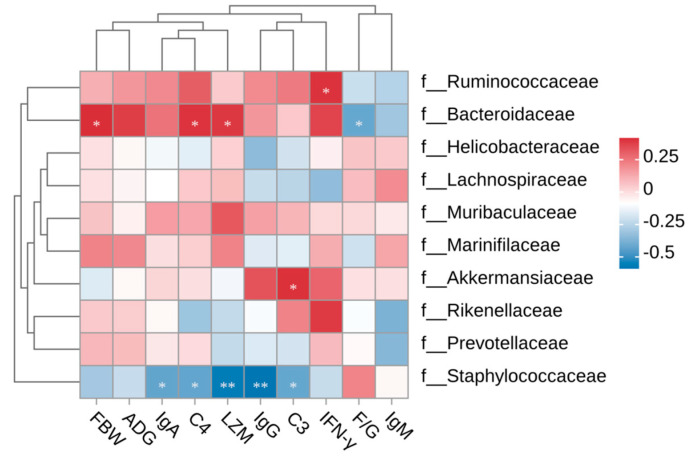
Spearman correlation heatmaps for cecal bacterial family. * *p* < 0.05; ** *p* < 0.01.

A clear association between the cecal microbiota at the family level and the FBW, ADG, F/G, and immunological indicators is apparent, as seen in Figure 7. IFN-γ levels showed a favorable correlation with *Ruminococcaceae*. FBW, Serum C4, and LZM levels had a positive correlation with *Bacteroidaceae*, whereas F/G showed a negative correlation. Serum C3 levels showed a positive correlation with *Akkermansiaceae*. Serum IgA, C4, LZM IgG, and C3 levels exhibited a negative correlation with *Staphylococcaceae*.

## 4. Discussion

### 4.1. Influence of RCIs on the Growth Performance of Mice

Many studies have demonstrated that the use of Chinese herbal medicine in animal feed improves growth, development, and production performance in animals. Xiao et al. [16] added Qi Weng-Huangbo powder into feed, which could enhance the ADG and FBW of mice. The supplementation of *Lycium barbarum* polysaccharides (LBPs) into feed significantly improved the weight gain of weaned piglets and reduced their feed-to-gain ratio. Furthermore, LBPs can significantly enhance the levels of IgG, IgM, and antioxidants [17]. *Eucommia ulmoides* peel extract can enhance immunoglobulin and antioxidant levels, and improve the cecal microbiota of white feathered broilers [15]. In this study, the supplementation of RCIs in feed improved the growth performance and feed efficiency of mice. The ADG of the mice in the middle RCI group reached the highest level (47.46%), while the F/G level reduced by 35.47%. This may be attributed to the estrogen-like effect of RCIs after entering the body. As an isoflavone, RCIs can bind to estrogen receptors to modulate the secretion of endogenous hormones, promoting the growth, development, and physiological activity of the animal.

### 4.2. Effects of RCIs on Serum Physiological and Biochemical Indicators of Mice

Routine blood indicators can be used to evaluate the health and growth condition of animals. White blood cells include granulocytes, monocytes, and lymphocytes, which participate in the body’s inflammatory response and immune defense by engulfing foreign substances, producing antibodies, repairing damage, and combating pathogen invasion. Furthermore, lysozyme can enhance the activation of neutrophils and the phagocytic activity of neutrophils and macrophages against bacterial pathogens [18]. This study suggested that RCIs increased the levels of WBCs, neutrophils, and lymphocytes, which is consistent with the findings of Criss [19].

Biochemical parameters are essential to assess the growth and health of animals. Total protein serves as an indicator of significant proteins in the body, including albumin and globulin. Albumin serves as a nutrient transporter and is used for tissue repair, energy provision, and the maintenance of serum osmolality. Blood urea nitrogen is the primary end product of protein catabolism, indicating the body’s nutritional state and the extent of protein metabolism. It is mainly excreted by the kidney and can reflect the health of the kidney. In this study, the total protein and albumin levels in the RCI groups were higher than those in control group, indicating that RCIs can increase protein catabolism and improve tissue repair capacity. Additionally, blood urea nitrogen levels showed no significant difference between the RCI and control groups, suggesting no toxicity of RCIs on the mouse kidney. Alkaline phosphatase indicates the absorption of calcium and phosphorus, which are crucial to bone growth. Creatine kinase is mainly found in skeletal muscle, heart, and cerebral tissue, and is an index of skeletal muscle injury. In this study, adding RCIs did not cause significant changes, indicating that RCIs have no effect on calcium and phosphorus absorption or on skeletal muscle damage. ALT and AST are essential aminotransferases for animals, regarded as significant indicators of hepatic damage. Generally, ALT and AST can sustain a dynamic equilibrium, but these parameters will markedly increase when pathological damage occurs in the liver [19,20]. In this study, the ALT and AST values did not vary much from those of the control group, indicating that RCIs have no negative impact on the liver.

### 4.3. Effects of RCIs on Immune Function of Mice

The enhancement of organ indexes indicates that the function of organs in the body is enhanced, which is beneficial to the body. The findings of this study indicated that the supplementation of RCIs increased the organ indexes of the heart, liver, spleen, and kidneys in mice, suggesting that RCIs may facilitate the development of these organs, contributing positively to the overall health of the mice. As the main immunoglobulins that protect the body against illness and viral invasion, IgA, IgG, and IgM are essential elements of humoral immunity. Reported investigations have demonstrated that dietary supplementation of probiotics and *Lycium barbarum* could increase IgA, IgG, and IgM levels in mice and weaned piglets [17,21]. Complement is a kind of immunological molecule that is particular to the innate immune system. The C3 and C4 components are the two primary components of the complement system, which serve an activation role [22]. As a foundational component of innate immunity, LZM has antibacterial and anti-inflammatory properties, as well as the capacity to promote the production of interferon, increase the activity of phagocytes, decrease the amount of waste nutrients, and improve digestion, as well as the ability to improve absorption [23,24]. IFN-γ is a cytokine that is generated primarily by T lymphocytes and killer cells. It is classified as an immunomodulatory cytokine that has the potential to mediate both humoral and cellular immunity, as well as enhance the immune system’s ability to defend itself [25]. The findings of this study indicated that the levels of IgA, IgG, C3, C4, LZM, and IFN-γ increased when adding 0.1% RCIs to the basal diet, which will contribute to improving the immune system and reducing the incidence of disease.

### 4.4. Microbiota Analysis

A healthy microbiota in the gut is particularly essential for developing an immune system and absorbing nutrients from foods. The abundance and diversity of microbial communities may be reflected in the alpha value. The evenness and variety of the species distribution can be reflected by the Shannon and Simpson indexes. The total number of species present in community samples can be estimated using the Chao index. The ACE index is used to estimate the population ASV. The α-diversity contributes to enhancing the growth performance of pigs [26]. The supplementation of 0.05% and 0.1% RCIs in the diet can improve the diversity of cecal microflora, and both the Chao 1 index and ACE index exhibited a growing trend. Therefore, RCIs might promote the growth performance of mice through increasing the abundance and variety of cecal microflora. *Bacteroides* in the gastrointestinal system may metabolize polysaccharides, hence augmenting the host’s nutrition absorption efficiency and facilitating the balance of the intestinal mucosa [27]. Furthermore, other studies have proven that the more *Bacteroides* in there are in the stomach, the higher the feed conversion efficiency [28]. We found that RCIs can increase the abundance of *Bacteroides*, suggesting its important role in the weight gain of mice.

In addition, the gut microbiota are closely related to the body’s immunity. The dietary supplementation of *Bifidobacteria* in mice raised their IgG, IgM, and IgA levels [29].

It has been reported that *Lactobacillus* and *Bifdobacterium* could reduce the release of inflammatory cytokines such as TNF-α and IL-6, as well as the anti-inflammatory cytokine IL-10, accompanied by significant boosts in IgAs/IgGs in a rat [30]. Another study has proven that *Bacteroides fragilis*-derived polysaccharides (PSAs) may stimulate CD4+ T cells, and these cells can produce IL-10, which may inhibit the occurrence of abscesses and other inflammatory reactions [31]. The *Firmicutes*/*Bacteroidota* ratio was inversely associated with IgA and LZM levels [32], which is consistent with our findings. The addition of 0.1% RCIs in the diet significantly reduced the *Firmicutes* population, while the richness of *Bacteroidota* increased, and IgA and LZM levels also increased. This phenomena may be explained by the activation of higher B-cells by *Bacteroidota*, such as *fragilis*, which may lead to elevated IgA concentrations, while RCIs can enhance LZM levels, hence reducing the *Firmicutes* to *Bacteroidota* ratio [33,34].

Furthermore, *Muribaculaceae* possess a substantial capacity to metabolize mucin glycans and dietary fiber polysaccharides, as well as to produce short-chain fatty acids (SCFAs) [35]. SCFAs are crucial for preserving intestinal health, regulating the immune system and inhibiting intestinal inflammation [36]. However, many members of the *Staphylococcaceae* family are pathogenic bacteria, including *Staphylococcus*, a common pathogen found in the intestines of cattle and poultry, which is able to cause many illnesses such as necrotizing enteritis, septicemia, and pneumonia [37]. In this study, supplementation of 0.1% RCIs can increase the abundance of *Muribaculaceae* and decrease the abundance of *Staphylococcaceae* pathogenic bacteria. In the Spearman correlation analysis, FBW, serum C4, and LZM levels were positively correlated and the F/G ratio was negatively correlated with *Bacteroidaceae*. Serum C3 levels were positively correlated with *Akkermansiaceae*. Serum IgA, C4, LZM, IgG, and C3 levels were negatively correlated with *Staphylococcaceae*. Therefore, it is speculated that the effects of RCIs on the regulation of host immune activity and growth promotion may be dependent on the changes in bacterial flora caused by RCIs. These results suggest a reciprocal regulation between dominant and deleterious bacterial families and immune cytokines after an RCI intervention in mice.

It is worth noting that *Akkermansiaceae* emerged in each RCI group. *Akkermansiaceae* employs intestinal mucins as its only carbon source and demonstrates favorable biological effects, including the enhancement of intestinal barrier integrity, modulation of immunological responses, and reduction of inflammation. Supplementation with some products that can increase the abundance of *Akkermansiaceae* to treat colitis and colon cancer has been proven effective [38]. *Akkermansiaceae* can also reduce the production of inflammatory cytokines and chemokines such as IL1α, IL6, and IL12A [39]. Accordingly, supplementation of RCIs may increase the abundance of *Bacteroidaceae*, *Muribaculaceae*, and *Akkermansiaceae*, simultaneously suppressing *Staphylococcaceae*, and thereby enhancing immunity and mitigating inflammation.

## 5. Conclusions

This research suggested that RCIs can significantly enhance both growth performance and immunological function and change the cecal microflora in mice. The recommended dietary concentration of RCIs was 0.1%. At a concentration of 0.1%, RCIs can significantly increase the daily gain and decrease the F/B ratio with no liver or kidney damage. Additionally, RCIs can increase the abundance of *Muribaculaceae* and *Bacteroidaceae* and reduce the abundance of *Staphylococcaceae*, which are closely related to the increases in some immunological indicators (IgA, C4, C3, and LZM). More importantly, RCIs promoted the growth of *Akkermansiaceae*, which can enhance the immune response and reduce inflammation. Therefore, there is an association between the changes in the cecal microflora and the improvement in the animal’s health. This study can provide a reference for the further development and utilization of RCIs as animal feed additives.

## Figures and Tables

**Table 1:** Ingredients and nutritional levels of baseline diet.

Ingredients	Content (%)	Nutrient Components	Content (g/kg)
Soybean meal, fish meal, brewer’s yeast powder	18	Crude protein	≥180
Vegetable oil	4	Crude fat	≥40
Bran	5	Crude fiber	≤50
Corn, Wheat	8	Coarse ash powder	≤80
VA, VD, VE, VB1, VB2, VB6, Pantothenic acid		Calcium	10~18
Calcium bicarbonate, iron, copper, manganese, zinc		Phosphorus	6~12
		Lysine	≥8.2
		Methionine and cystine	≥5.3

**Table 2:** Routine blood indexes of mice.

Items		Groups		
	CK	L	M	H
WBC (10^9^/L)	3.15 ± 0.57	3.91 ± 0.17 *	4.61 ± 0.16 **	2.85 ± 0.08
Neu# (10^9^/L)	0.41 ± 0.04	0.78 ± 0.11 **	0.53 ± 0.12	0.43 ± 0.16
Lym# (10^9^/L)	2.58 ± 0.6	2.56 ± 0.67	3.36 ± 1.19 **	2.4 ± 0.12
Mom# (10^9^/L)	0.02 ± 0.01	0.03 ± 0.01	0.06 ± 0.03 *	0.02 ± 0.01
Neu (%)	14.63 ± 3.48	20.50 ± 5.79	11.60 ± 2.52	14.70 ± 5.22
Lym (%)	84.37 ± 3.59	78.30 ± 5.54	87.10 ± 1.71	84.20 ± 5.45
Mom (%)	0.90 ± 0.36	1.10 ± 0.46	1.20 ± 0.26	0.90 ± 0.36
RBC (10^12^/L)	9.94 ± 1.01	10.00 ± 0.72	9.64 ± 0.73	9.75 ± 0.45
HGB (g/L)	160.33 ± 14.15	159.67 ± 3.79	154.67 ± 13.01	158 ± 8.19
HCT (%)	47.97 ± 4.31	48.03 ± 1.66	46.63 ± 3.21	47.07 ± 1.27
MCV (f L)	48.37 ± 1.38	48.13 ± 2.76	48.4 ± 0.62	48.3 ± 1.66
MCH (p g)	16.13 ± 0.38	16.00 ± 1.05	16.03 ± 0.25	16.20 ± 0.62
MCHC (g/L)	333.67 ± 1.15	332.67 ± 3.79	331.33 ± 9.61	335.33 ± 8.08
RDW-CV (%)	14.17 ± 1.36	12.67 ± 1.53	13.27 ± 0.55	12.33 ± 0.35
RDW-SD (f L)	26.20 ± 1.95	23.37 ± 2.84	24.70 ± 1.06	23.10 ± 1.06
PLT (10^9^/L)	831.33 ± 121.70	688.67 ± 188.39	1048.33 ± 128.85	824.00 ± 129.22
MPV (f L)	5.50 ± 0.26	5.63 ± 0.42	5.63 ± 0.15	5.60 ± 0.30
PDW (f L)	15.57 ± 0.12	15.5 ± 0.35	15.27 ± 0.12	15.43 ± 0.12
PCT (%)	0.46 ± 0.08	0.39 ± 0.10	0.59 ± 0.09	0.46 ± 0.05
WBC (10^9^/L)	16.13 ± 0.38	16.00 ± 1.05	16.03 ± 0.25	16.20 ± 0.62

A basic diet was given to the mice in the control group and the L, M, and H groups were fed with 0.05%, 0.1%, and 0.2% RCIs in a basic diet, respectively. * *p* < 0.05; ** *p* < 0.01.

## Data Availability

The original contributions presented in this study are included in the article. Further inquiries can be directed to the corresponding authors.
